# Vaccine-Induced Cellular Immunity against *Bordetella pertussis:* Harnessing Lessons from Animal and Human Studies to Improve Design and Testing of Novel Pertussis Vaccines

**DOI:** 10.3390/vaccines9080877

**Published:** 2021-08-07

**Authors:** Anja Saso, Beate Kampmann, Sophie Roetynck

**Affiliations:** 1 The Vaccine Centre, Faculty of Infectious and Tropical Diseases, London School of Hygiene & Tropical Medicine, London WC1 7HT, UK; Beate.Kampmann@lshtm.ac.uk (B.K.); sroetynck@mrc.gm (S.R.); 2 Vaccines and Immunity Theme, MRC Unit, The Gambia at London School of Hygiene & Tropical Medicine, Banjul P.O. Box 273, The Gambia

**Keywords:** *Bordetella pertussis*, whooping cough, T-cells, B-cells, vaccination, immunity, correlate-of-protection, antigen-specific, epitope, colonisation

## Abstract

Pertussis (‘whooping cough’) is a severe respiratory tract infection that primarily affects young children and unimmunised infants. Despite widespread vaccine coverage, it remains one of the least well-controlled vaccine-preventable diseases, with a recent resurgence even in highly vaccinated populations. Although the exact underlying reasons are still not clear, emerging evidence suggests that a key factor is the replacement of the whole-cell (wP) by the acellular pertussis (aP) vaccine, which is less reactogenic but may induce suboptimal and waning immunity. Differences between vaccines are hypothesised to be cell-mediated, with polarisation of Th1/Th2/Th17 responses determined by the composition of the pertussis vaccine given in infancy. Moreover, aP vaccines elicit strong antibody responses but fail to protect against nasal colonisation and/or transmission, in animal models, thereby potentially leading to inadequate herd immunity. Our review summarises current knowledge on vaccine-induced cellular immune responses, based on mucosal and systemic data collected within experimental animal and human vaccine studies. In addition, we describe key factors that may influence cell-mediated immunity and how antigen-specific responses are measured quantitatively and qualitatively, at both cellular and molecular levels. Finally, we discuss how we can harness this emerging knowledge and novel tools to inform the design and testing of the next generation of improved infant pertussis vaccines.

## 1. Introduction

### 1.1. The Story so Far

*Bordetella pertussis* (*Bp*) is a gram-negative bacterium that causes pertussis (‘whooping cough’), an acute respiratory tract infection which disproportionally affects young children, particularly unimmunised infants [1,2]. Up until the early twentieth century, whooping cough was one of the most important causes of mortality in children under 5 years; the introduction of the first whole-cell pertussis vaccine (wP), consisting of detoxified killed whole *Bp*, within national immunisation programmes in the 1940–50s was therefore hailed as a success story [3,4]. However, due to its perceived neurological side effects and broader reactogenicity, caused by the multiple bacterial antigens present, including highly immune-stimulatory cell wall component lipooligosaccharide (LOS) and other virulence factors, vaccine uptake subsequently began to decline [5]. As such, a safer alternative was developed, the acellular pertussis (aP) subunit vaccine, which was shown to confer equal protection against severe symptomatic disease [6]. Several aP vaccines are available differing by the number of *Bp* antigens that they contain, their dose, as well as their formulation; currently licenced variations contain alum adjuvant and between one to five purified, stabilised, and chemically or genetically modified (detoxified) pertussis antigens, including pertussis toxin (PT, at least), filamentous haemagglutinin (FHA), pertactin (PRN), and fimbrial proteins 2 (FIM2) and 3 (FIM3).

From the 1990s, therefore, aP began to be rolled out globally by the World Health Organisation (WHO) and was incorporated into the primary immunisation schedule of most high-income countries (HICs) [7,8]. Despite high vaccine coverage rates (especially in HICs), however, there has been a resurgence in *Bp* disease worldwide and it remains a primary cause of vaccine-preventable death [9,10]. Recent models have indicated that there were 24.1 million pertussis cases and 160,700 deaths in children younger than 5 years worldwide in 2014, with the highest burden in sub-Saharan Africa, although these are estimates given the paucity of pertussis epidemiological data to date [11]. In many low-to-middle-income countries (LMICs), the suboptimal pertussis control is thought to be primarily due to limited access to vaccines and inadequate healthcare resources, with poor diagnostic tools [12,13,14]. In HICs, the situation is more complex, and several reasons have been postulated for the increased reporting, including improved diagnostics, enhanced surveillance, changes in immunisation strategy and administration schedules, differences in vaccine composition and immune responses induced, and antigenic variation in the circulating strains of *Bp* due to vaccine selection pressure, reviewed extensively elsewhere [4,10,15,16].

The most plausible explanation is the switch in infant primary immunisation from wP to aP vaccines, which has been linked to less effective protection (particularly against colonisation, infection and transmission) and waning immunity [10,17,18,19,20,21]. The data to support this hypothesis primarily stems from both epidemiological studies and animal infection/transmission models. Infection in predominantly aP-vaccinated settings peaks in two age groups: (1) infants too young to have received the primary immunisation schedule and who should, therefore, benefit from herd immunity, suggesting that sterilising immunity is not achieved through immunisation; colonisation (often asymptomatic) can thus still occur, facilitating bacterial transfer to vulnerable, unprotected cohorts (2) adolescents and adults, although the clinical presentation is less severe, indicating that different specific long-term immune memory is elicited by the vaccines, with pertussis-specific immunity waning more rapidly following aP vaccination. Observational studies have shown that older children primed with aP compared to wP vaccines in infancy had a 2-to 5-fold greater risk of pertussis diagnosis [22,23,24]. Moreover, a US case control study demonstrated that, among adolescents who have only received DTaP vaccines in childhood, vaccine effectiveness following Tdap booster was 68.8% during the first year after vaccination, rapidly declining to 8.9% by ≥4 years after vaccination [25]. Nevertheless, even wP vaccines do not seem to establish as effective or long-lasting immunity as natural infection [26]. Pertussis outbreaks in these two cohorts have, therefore, led to the introduction of multiple boosters in some countries (particularly in the primary school and adolescent age groups) and routine maternal vaccination in pregnancy to protect the new-born in early life, prior to their first priming vaccine dose [27,28].

### 1.2. Differences in Vaccine Composition and Host Immune Responses Go Hand-in-Hand

Differences between vaccines are multifactorial and interrelated. Key contributing factors are the underlying qualitative and quantitative immunological mechanisms that mediate efficacy and longevity of vaccine-induced protection, particularly at the respiratory mucosal interface. Animal models have shown that memory CD4+ T-cells of T-helper (Th-)1 and Th17 phenotype facilitate long-term protection, which are elicited by natural infection as well as immunisation with wP [29,30,31,32]. In contrast, aP vaccination is associated with a predominant Th2 response in humans [33,34,35,36,37]. Beyond Th-cell polarisation, other qualitative changes in cellular responses may result in suboptimal and/or shorter efficacy [21,38,39,40,41]. For instance, given that wP vaccines appear to prevent colonisation in animal models, they may induce immune responses that home to and/or act more effectively at the mucosal interface compared to aP vaccines [17,31,42,43,44].

These mechanisms are, in part, shaped by the choice of adjuvant in either vaccine. The more durable priming upon wP vaccination might especially be due to the highly immunostimulatory LOS, operating as an adjuvant by activating innate immune cells, for example, via toll-like receptor (TLR-)4 [6,26,45]. By contrast, aP vaccines primarily contain the less reactogenic adjuvant, alum, which potentially contributes to the limited duration of effective protective immunity against pertussis. Other factors related to vaccine composition may further influence the differential immune responses. One example is the chemically detoxified PT contained in the aP vaccine, which may have altered antigenicity and could potentially impact vaccine efficacy [1,16]. Furthermore, given that the wP vaccine contains >3400 open-reading frames, whereas the aP vaccine includes only five or fewer *Bp* proteins, it is likely that the former elicits a broader reactivity and targets additional surface antigens, some of which might be of particular relevance and linked to superior vaccine performance [46,47]. This potentially underlies differences observed in the breadth of antibody responses and/or functional antibodies induced: primarily PT-neutralising compared to a combination of neutralising, agglutinating and opsonising antibodies following aP and wP vaccines, respectively [48,49,50]. However, to date, the extent and targets of T-cell immunity in the context of natural infection, clinical disease or vaccination have not been comprehensively defined. Of note, comparisons of vaccine composition (and immune responses elicited) are hampered by the heterogeneity of aP vaccine formulations, immunisation strategies and administration schedules.

Beyond this, antigenic drift may potentiate reduced vaccine effectiveness and waning of *Bp* immunity, particularly in settings with widespread aP use [46,51,52,53]. Novel circulating *Bp* strains have evolved, containing mutations and/or deletions of key epitopes or antigens; this is secondary to pathogen adaptation to natural or, most likely, vaccine selection pressures, with subsequent pathogen escape [46]. Examples include strains with polymorphisms of the PT gene (leading to enhanced production of this protein), or which lack the PRN gene (demonstrating increased fitness and/or prolonged infection times, given that PRN is a key target of opsonising antibodies), primarily in the context of aP-containing immunisation schedules [16,48,54,55]. However, data on the impact of these mutations and similar genetic modifications on vaccine-mediated efficacy, particularly in settings of PRN-deficient *Bp* strains are conflicting and further studies are urgently required [56]. Finally, whether specific T-cell immune pressure has resulted in the observed genetic variability of these strains is yet to be fully elucidated [46,53].

### 1.3. Evidence for the Importance of Cellular-Mediated Immunity

No clear correlate-of-protection (CoP) against pertussis disease has been identified which could inform vaccine development and licensure [57,58]. Pertussis-specific serum antibody response is important in limiting severity of disease [59] and to some extent infection, however, the currently available antibody assays are insufficient to represent a CoP [60,61,62]. Cell-mediated immunity (CMI) is increasingly thought to play a key role in both vaccine efficacy and longevity of protection, via effector mechanisms as well as influence on magnitude, quality and longevity of the broader immune response [41,63,64]. High antibody levels are elicited by both vaccines, although less is known about potential qualitative differences in antibody responses, which may be T-helper cell related. Vaccine-induced protection against disease persists even after circulating antibody titres have waned and/or are absent at the time of challenge [6,21,65]. Repeated pertussis immunisation of B-cell knockout (BKO) mice resulted in partial protection. Moreover, mice depleted of CD4+ T-cells after immunisation but before aerosol challenge, who therefore had normal levels of specific antibodies, were not optimally protected [66]. Complete protection was reconstituted by transfer of pertussis-immune B-cells; reconstituted BKO mice had little if any detectable anti-pertussis antibodies [66]. Coupled with the dual nature of *Bp* as an intracellular and extracellular pathogen, these findings already suggest a key role for (primarily CD4+) T-cells and B-cells in protective immunological memory, with both cell types providing significant functions other than specific antibody production [36,41,63,64,65,66,67]. Furthermore, emerging evidence from studies of both pertussis and other respiratory pathogens indicates that the local tissue-resident memory T-(TRM) cells that accumulate in respiratory tissue following mucosal infection may be crucial for long-term immunity [42,43]; elucidating their phenotype and functional capacity in the context of vaccination is therefore critical.

**Figure 1:**
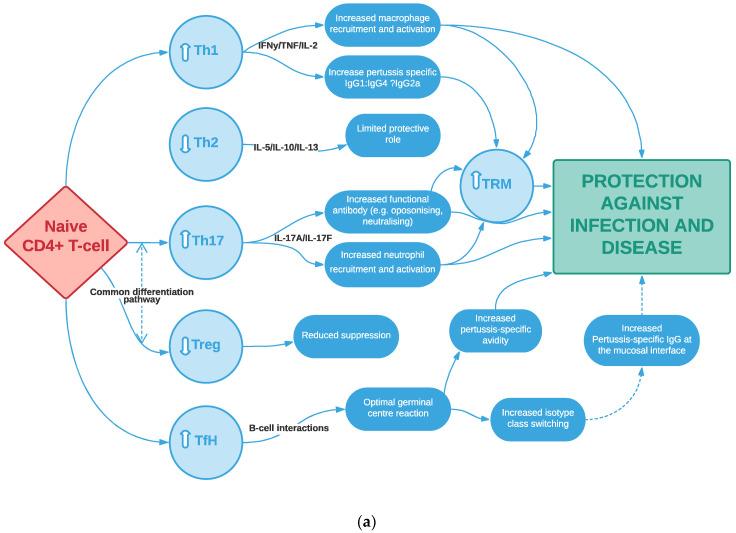

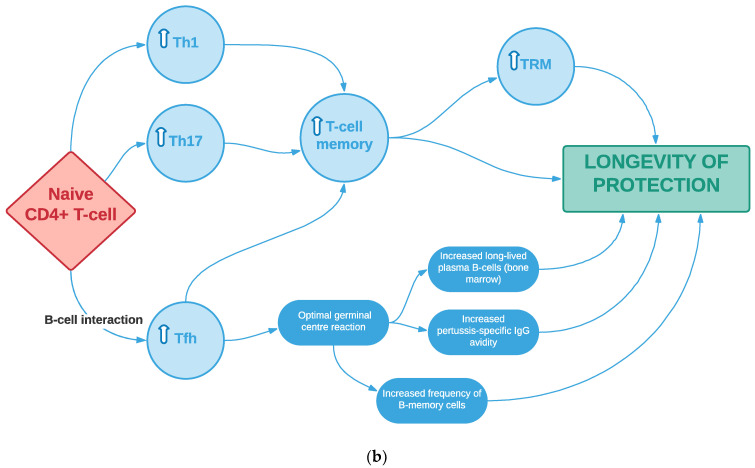
Schematic summary of the hypothesised immunological differences between the two infant pertussis vaccines, including their impact on: (**a**) protection against pertussis infection and disease; (**b**) longevity of protection. Shown are systemic T-helper cell responses induced by the whole-cell pertussis infant vaccine and how they might compare to acellular pertussis immunisation (indicated by white arrows); proposed mechanisms are antibody/B-cell dependent and independent. Ig, immunoglobulin; Th, T-helper; Tfh, T-follicular helper; Treg, T-regulatory; TRM, tissue-resident memory cells.

This review will summarise current knowledge on the nature, frequency and breadth of antigen-specific cellular responses induced following childhood pertussis immunisation. In particular, we will address how these mechanisms shape the ability of aP compared to wP vaccines to protect against infection/transmission and to induce long-lived immune memory. Our focus will be on the (pertussis-specific) T-cell compartment, as measured in peripheral blood and/or at the level of the respiratory mucosa, including its interrelationship with B-cell-mediated responses and innate immunity (Figure 1a,b).

## 2. What Have We Learned about Vaccine-Induced T-Cell-Mediated Immunity from Animal Models?

### 2.1. The Mouse Model

#### 2.1.1. Systemic Responses

Seminal mouse studies by Mills *et al.* first highlighted the critical role of T-cells in the direct protection against *Bp* infection, by showing that mice able to clear the infection generated robust antigen-specific T-cell responses in the absence of a detectable serum antibody response [29,68]. Furthermore, adoptive transfer of spleen cells or purified CD4+ T-cells (but not CD8+) from convalescent mice conferred protection against *Bp*-respiratory challenge in nude or irradiated mice resulting in bacterial clearance from the lung, [32,63,69]; these cells were polarized to Th1 and Th17 phenotypes, although the greatest protection was observed when both cell-types were transferred [67]. Injection of serum from protected animals, however, only marginally reduced bacterial loads [29,67,68].

Subsequent mechanistic studies using mice defecting in individual cytokines and/or receptors confirmed the important role of Th1 and Th17-cells in recruiting phagocytes to the respiratory tract, bacterial clearance and protection against subsequent challenge [29,67,69,70,71,72]. Infection in IFN-γ−/− and IFN-γ receptor −/− mice led to atypical or disseminated disease, while IL-17−/− mice demonstrated significantly increased *Bp* load in lung samples, reduced CXCL1 production and impaired neutrophil recruitment post-*Bp* challenge [67,69,73]. *Bp* virulence factor, adenylate cyclase toxin, was shown to activate caspase-1/NLRP3 inflammasome, thereby promoting IL-1β production by murine macrophages or dendritic cells and subsequent bacterial clearance [74]. *Bp* pathogenesis was significantly exacerbated in IL-1R type I-defective mice [74,75]. In contrast, primary infection with *Bp* was not significantly different between IL-4−/− and wild-type mice, suggesting that Th2-CMI is not essential for protection [67].

Beyond this, the mouse respiratory infection model has proven a useful tool for the evaluation of vaccine-induced immunity, since bacterial clearance from lungs following exposure to *Bp* correlates with vaccine efficacy [70,71,76,77]. After exposure to a *Bp* respiratory challenge, convalescent mice and those immunised with wP eliminated the bacterial infection significantly faster than mice immunised with aP [63]. Consistent with natural infection, effective pertussis immunisation in mice has been shown to depend on induction of CMI, with no protection established in mice lacking all T-cells or CD4+ T-cells [66]. Furthermore, polarisation of Th-cells occurs, determined by infant priming [63,67]. Th1/Th17 subsets coordinate wP-induced immunity [45,67,71] with a larger contribution by IFN-γ-secreting cells compared to Th17. In contrast, aP immunisation elicits Th2/Th17 responses [67,71,78]; however, IL-17A plays an essential role, with failure of protective immunity in IL-17A defective mice, while IL-4 is unnecessary for bacterial clearance [67,78].

#### 2.1.2. Mucosal Responses

In addition to characterising systemic immune responses, the murine model has enabled mechanistic studies of CMI at the mucosal level, in both the upper and lower airways. Following *Bp* infection, mice express IL-17, IL-6, and IL-8 homologs in the lungs consistent with a local Th17 response [79].

Antigen-specific tissue resident memory cells (TRMs) have garnered significant interest recently as they are increasingly thought to be critical in establishing protection against reinfection at the mucosa and long-term immune memory against different pathogens [26,43,80]. Specifically, IL-17- and IFNγ-secreting CD69+CD4+ TRM cells were shown to accumulate in the lungs of mice during pertussis infection [81]. The same cell phenotype significantly expanded in both lungs and nasal tissue after *Bp* challenge and reinfection of convalescent [42,43,81,82] or wP- but not aP-immunised mice [83]. Sphingosine-1-phosphage receptor agonist FTY720 (fingolimod) blocks T- and B-cell migration from lymph nodes to the circulation without impairing their activation; FTY720 administration to mice during primary infection or wP immunization resulted in reduced homing of central memory T- and B-cells to the lungs, which was further associated with impaired *Bp* clearance. Conversely, treatment with FTY720 before re-challenge did not affect the local expansion of TRM cells and hence the associated rapid clearance of the secondary infection. Therefore, wP vaccination of mice elicited protection against lung and nasal colonisation whereas aP immunisation failed to protect in the nose. Similarly, adoptive transfer of lung CD4+ TRM cells from convalescent or wP-immunised mice conferred protection in naïve mice [83]. Previous infection, however, induced the most persistent protection against nasal colonisation and this correlated with potent induction of nasal tissue TRM cells, especially those secreting IL-17 [42,81,83]. Expansion of the TRM population has been observed following select next-generation vaccines, including intranasal live-attenuated BPZE1, outer membrane vesicle (OMV) vaccines, and intranasal administration of aP vaccine with TLR9 and stimulator of interferon genes (STING) agonists (Table 1).

As well as CD4+ TRM cells, pulmonary γδ T-cells may be another key player in mucosal immunity. They enable both an early source of innate IL-17, thereby promoting antimicrobial peptide production, as well as adaptive immunological memory against *Bp*, analogous to conventional αβ T-cells, through antigen-specific Vγ4 cells. γδ T-cells with a TRM phenotype (i.e., CD69+CD103+) were shown to expand in the lungs during infection with *Bp* and proliferate rapidly after rechallenging convalescent mice [82]. Their role in vaccine-induced protection is still being explored.

Finally, although these findings confirm the contribution of local mucosal responses to effective bacterial clearance and prevention of colonisation, to date a detailed characterisation of transmission dynamics has not been possible in mice. Nevertheless, Scanlon *et al.* recently reported the first study of *Bp* transmission in murine neonates, which may prove a valuable tool in future mechanistic research on vaccine-induced immunity [84].

### 2.2. The Baboon Model

#### 2.2.1. Colonisation and Transmission Studies

Comparison of published murine data is difficult, given the heterogeneity in approaches and methodology. Various mouse strains (e.g., BALB/c vs. C57Bl/6), immunisation routes (e.g., intraperitoneal vs. intracerebral), vaccine doses, compositions and regimens have been studied; moreover, different infectious challenge doses of varying *Bp* strains were administered in different suspension volumes, either directly into mouse nares, or as inhaled aerosol [48]. Most importantly, the murine challenge model does not accurately recapitulate the natural disease course in humans. Instead, the baboon model has been increasingly used to study both pertussis disease pathology and vaccine-induced immune responses [85]. The baboon displays similar systemic symptoms (lymphocytosis, hypoglycaemia) and respiratory characteristics (heavy respiratory colonization, paroxysmal cough, >2 weeks, aerosol transmission) to humans. Furthermore, immunological mechanisms vary between mice and humans, for example TLR distributions and IgG subclasses [86,87,88].

A series of studies in the baboon demonstrated that aP vaccination provides excellent protection against severe disease but does not prevent colonisation, either following direct challenge (*Bp* aerosol) or infection due to transmission (between vaccinated and naïve subjects) [17,30,31,86,89,90,91]:

(a) Following direct challenge with *Bp* aerosol, wP- and aP-vaccinated baboons displayed similar initial nasopharyngeal colonisation, although the former group cleared infection significantly faster than naïve and aP-immunised animals. Neither wP-vaccinated, aP-vaccinated, nor convalescent baboons showed signs of severe respiratory or systemic disease.
(b) To evaluate whether vaccination prevents pertussis infection by natural transmission, two aP-vaccinated animals and one unvaccinated baboon were housed together with a directly challenged, unvaccinated baboon (who subsequently became infected). All animals were colonised by 7–10 days, with no significant differences in peak levels and kinetics of colonisation between naïve and aP-immunised baboons.
(c) To confirm that aP-vaccinated baboons are not only capable of being colonised but can subsequently also transmit *Bp* to naïve contacts, aP-vaccinated animals were challenged with *Bp* and placed in separate cages. After 24 h, a naïve animal was added to each cage; both were infected by transmission from their aP-vaccinated cage mates.

#### 2.2.2. Systemic Responses

Characterisation of the phenotype of the baboon cellular compartment demonstrated similar Th1/Th2/Th17 polarisation to the murine model. Long-lived *Bp*-specific Th17 and Th1-memory-cells were observed in convalescent animals >2 years post-infection suggesting a role in immunological memory to pertussis. Similarly, CD4+ T-cells from wP-vaccinated animals secreted IFN-γ but no IL-5; IL-17 secretion was between levels for naïve and convalescent baboons, suggesting that wP vaccination induces similar, albeit weaker, Th1 and Th17-memory responses to natural infection. aP-vaccinated baboons, however, demonstrated a significant IFN-γ (similar to wP) and IL-5 response, consistent with skewing toward Th1/2 memory, but no significant increase in IL-17 secretion, contrasting findings in mice [30,31].

#### 2.2.3. Mucosal Responses

At the mucosal level, primary pertussis infection is characterised by a transient increase in IL-17, IL-6, IL-8 and other cytokines and chemokines associated with Th17 response and neutrophil activation in baboon nasopharyngeal samples [30,91]; analysis of the mucosal immune milieu (including cytokines and pertussis-specific antibody) following aP versus wP vaccines is awaited. These findings suggest a plausible explanation for the differences observed in the strength and persistence of protective immunity induced by the aP or wP vaccines in humans, contributing to the recent pertussis outbreaks [16,31,88,91]. Nevertheless, the baboon model has disadvantages, not least the low numbers that can be included in one experiment simultaneously, the time taken to breed each baboon (gestation time of ~6 months) and poor availability of primate cell-markers.

### 2.3. Other Animal Models

Another promising model is the pig, given that its immune system more closely resembles that of humans (when compared to mice), particularly TLR distributions and responsiveness, Th17 characteristics and breast milk composition. Moreover, porcine systemic and mucosal immune compartments are easily accessible, and many major markers are available for use in porcine cells [92,93]. Pigs can be challenged with *Bp* and display a wide range of respiratory and systemic symptoms, consistent with humans. Viable bacteria can be re-isolated from bronchoalveolar lavage specimens and lung lesions for >10 days after infection.

## 3. What Have We Learned about Vaccine-Induced T-Cell-Mediated Immunity from Human Studies?

Results from human studies, across all age-groups, demonstrate the important contribution of persistent pertussis-specific CMI to the long-term protection achieved by both natural infection and vaccination; this may either be through its own effector mechanism or by helping the antibody response [36,40,41]. T-cell responses, particularly of CD4+ memory phenotype, specific for the vaccine components are induced that increase progressively over the course of the primary infant schedule and are maintained for at least a few years, in contrast to waning antibody titres [94,95].

Studies are heterogenous, however, particularly in their methodology and length of follow-up, measuring different age groups, immunisation schedules and T-cell parameters, and further compounded by variations in vaccine formulations across different settings [16,41]. Nevertheless, several key ideas emerge from the literature, albeit sometimes conflicting:

### 3.1. Distinct Patterns of Cellular Immune Responses Are Induced by aP vs. wP and Are Largely Consistent with the Th1/Th2 Polarisation Observed in Animal Models

Natural infection and wP vaccination induce Th1-dominated responses corroborating findings in animal models [33,36]. Moreover, initial studies comparing magnitude of CMI at different time points following aP vaccinations (primary or booster) versus either natural infection or wP in children found an equally potent, and in some cases higher, response [94,96,97,98,99,100,101]. In both aP-vaccinated and naturally infected infants, cytokine secretion profiles demonstrated preferential induction of IL-2- and IFN-y-producing Th1 cells and only low production of IL-10 [33,94,97]. In a separate study, following the primary DTaP vaccine series, most participants mounted a positive proliferative T-cell response to the PT and PRN antigens, with fewer positive responses to FHA and FIMs. One month after booster DTaP vaccination (age 16 to 19 months), the cytokine profile was consistent with Th1 skewing: a significant increase in IFN-γ production with the PT and FIM antigens, a significant increase in IL-2 production with the PT, FHA, and PRN antigens, and lack of significant IL-4 secretion with any of the antigens [102].

Furthermore, several Dutch studies have shown that replacement of wP by aP combination vaccines in infancy enhanced pertussis immune responses in the short term until 6 years of age, i.e., following the Dutch primary immunisation schedule (4 doses in the first year of life) and then up to 2 years after the fifth DTaP booster given at preschool age [39,98,99]. Specifically, DTaP booster vaccination induced more pertussis-specific CD4+ effector memory cells in preschool 4-year-old aP-primed compared to wP-primed children; in contrast to previous studies, however, both Th1 and Th2-profiles were detected [99,103].

Other groups more recently have similarly documented a mixed Th1/Th2 or a Th2-skewed response in infant, child, adolescent and even in adult cohorts who have been primed with aP compared to wP vaccines [36,37,104,105]. Ryan *et al.* examined cellular responses in children immunised with a range of different aP and wP vaccines, concluding that the mechanism of protection generated following aP immunisation was more heterogeneous, involving a mixed Th1 and Th2 cellular response [36]. These findings were observed not only in response to the key pertussis vaccine antigens, but also to heterologous co-administered tetanus toxoid. This suggests that the early cytokine profile is modulated by the type of pertussis vaccine formulation [34,106]. Furthermore, differences in innate cells and inflammatory signalling pathways triggered by wP versus aP vaccines may contribute to the T-cell polarisation [107].

These differences in data may be explained by the heterogeneity of studies and their methodology, as discussed previously. Van der Lee *et al*, however, attempt to reconcile these conflicting findings by arguing that after 6 years of age, specifically following the preadolescent Tdap booster vaccination (at 9 years), there is a shift in humoral and cellular immunity [39]. While Th2 responses were similar between the aP- versus wP-primed groups, preadolescents primed with wP vaccines during infancy had the more favourable Th1-dominated immune response compared to aP-primed preadolescents for at least 1 year after the booster vaccination. Polarisation toward a Th2 profile in aP-primed preadolescents was caused by a decreased production of the Th1 cytokines (IFN-γ), and not by a higher Th2 cytokine (IL-13) production. In keeping with this data, the shift in vaccine-specific Th-cell pattern observed throughout childhood may reflect the developmental processes occurring within the overall adaptive immune compartment. This global Th2 skewing may be overcome by the unique composition of wP (i.e., presence of LOS) [104].

Characterisation of vaccine-induced antigen-specific IgG subclass responses following different pertussis priming schedules further supports Th-subset skewing [38,108]. The IgG4 proportion was found to be significantly higher in aP- compared with wP-primed children. This subclass does not bind complement and leads to a suboptimal inflammatory response, with impaired phagocytosis and antimicrobial defence, hence this may contribute to reduced protection against pertussis subsequently. These observations are consistent with murine data, given that IgG4 is functionally analogous to mouse IgG1 [26]. By contrast, wP infant priming results in better opsonisation, phagocytosis, and complement-mediated killing through the preferential induction of IgG1, despite subsequent aP boosters [38,39,49].

### 3.2. Th-Cell Polarisation Established in Infancy Persists Even if Subsequent aP Boosters Are Given in Adolescence and Adulthood

The current consensus is that childhood aP versus wP vaccines induce functionally different patterns of T-cell responses to pertussis that remain skewed, even upon boosting (by natural infection due to high pertussis circulation or following a single aP/dTaP vaccine dose), up to several years or decades after the original priming [37,103,109]. aP-primed children 4-years of age showed high Th1 and Th2 responses, more effector memory and terminally differentiated CD4+ T-cells that remained unchanged or even decreased upon the preschool booster, whereas those in wP-primed children were low but increased upon a preschool booster vaccination [98,99,103]. Later in childhood, Th1 and Th2 cytokine levels, and therefore Th1/Th2 ratios, remained unchanged before and after the preadolescent booster vaccination in both groups of children [101]. This polarisation was still observed even in adolescents primed with repeated aP vaccines during infancy [37,39,109]. Some studies, however, have challenged these findings [110].

Of note, there is minimal data addressing potential issues around mixing aP/wP vaccine formulations throughout childhood, and the subsequent impact on effectiveness or duration of protection [111].

### 3.3. Aside from Th Skewing, Infant Vaccine Type May Influence the Strength and/or Duration of the Cellular Response

A recent comprehensive study demonstrated that adolescents originally primed with aP have decreased capacity to respond to a booster immunisation *in-vivo* and diminished proliferative capacity of T-memory cells *in-vitro*, potentially suppressed by a regulatory cell population [109]. Similarly, in a pre-adolescent cohort, although the time since the last booster vaccine was significantly longer for wP-versus aP-vaccinated individuals, their proliferation capacity in response to antigenic stimulation was comparable; more children also had a detectable cytokine response after wP compared to aP-vaccination [101]. A further study documented persistence of T-cell memory against PT only in a minority (36.8%) of children 5-years after aP priming. Differences in T-cell responses and potentially long-term protection were demonstrated even between aP vaccine types, possibly explained by variations in PT inactivation processes or excipient/adjuvant formulations [112]. These findings corroborate recent epidemiological studies at the immunological level, indicating that infant vaccination with wP induces longer lasting immunity than vaccination with aP-vaccines. Indeed, repeated booster doses of the aP vaccine in children primed with the same vaccine type has been shown to result in limited memory subset expansion and/or progressively shorter duration of protection against disease [49,103,112].

### 3.4. Th17 Cells, Particularly of Tissue-Resident Memory Phenotype, Potentially Play an Important Role in Pertussis Vaccine-Induced Immunity, Consistent with Animal Studies

There is indirect evidence of asymptomatic transmission of *Bp* from aP-vaccinated to naïve individuals [20,113,114,115]. To date, however, little is known on human vaccine-induced mucosal immune responses, the contribution of respiratory TRMs, particularly of Th1 and Th17 phenotype, to protection against infection/disease and how this may vary between vaccine types [42,116].

One previous study observed low systemic Th17 responses in both wP- and aP-primed preadolescents, proposing that these responses may be far less pronounced upon vaccination in humans than in animal models [37]. More recently, however, Antunes *et al.* were the first to show that induction of antigen-specific Th17-cells following a pertussis booster vaccine in adolescents was determined by their immunisation type given in infancy, confirming results in the baboon (but not in mice). Applying a transcriptomics systems vaccinology approach, they proposed that in comparison to wP priming, aP immunisation results in higher production of IL-1 and IFN-β; this may in turn modulate the effect and magnitude of TGF-β production which can block Th17 differentiation in aP donors [109].

As discussed previously, Th17 is important in clearance of infection at the level of the mucosa, through both direct and indirect mechanisms [26,117,118]. Indeed, Th17 cells are key in promoting polymeric-IgR-mediated transport of secretory-IgA (sIgA) and IgM into the airway [119]. *Bp* leads to potent pertussis-specific sIgA production in nasal secretions which inhibits adherence of *Bp* to human respiratory epithelial cells, persisting for several months after onset of symptoms [120]. Currently, however, there is conflicting and limited evidence on the effect of mucosal IgA (and IgG) on pertussis disease severity, infection/colonisation and vaccine-induced protection in both animal models and human studies [44]. Moreover, little is understood about the kinetics of cytokines, particularly those related to development and maintenance of Th17 response, and sIgA induced at the mucosa following differential pertussis priming schedules. Knowledge is notably sparse in younger paediatric cohorts, hindered by challenges in obtaining respiratory mucosal samples, specifically lymphoid tissue, in this age-group.

### 3.5. Other T-Cell Subtypes May Play a Key Role in Vaccine-Induced Immunity

Most human vaccine studies have focussed on Th1/2 and, more recently, Th17 cellular profiles. But other T-cell types may contribute to the differences in vaccine-induced immunity and therefore require further attention. In addition to confirming the critical role of pertussis-specific Th17 cells, Antunes *et al.* also demonstrated the potential importance of (a) IL-9 secreting T-cells in vaccine-mediated protection and (b) regulatory cells that may inhibit T-memory-cell proliferation following pertussis booster vaccine in aP compared to wP-primed infants [109]. This suggests the need to explore the potential contribution of antigen-specific Th9 and T-regulatory(T-reg) cells in future studies. On the other hand, previous groups found similarly low IL-10 secretion in both vaccine groups, a key cytokine often involved in coordinating the T-reg response [37].

Another important T-cell type are T-follicular-helper (Tfh)-cells that bridge the T- and B-cellular compartments and are crucial to establishing/maintaining long-lasting highly specific memory [121,122]. Tfh promote survival, affinity maturation, and class switch recombination of B-cells at the germinal centre (GC), as well as generation of B-memory (Bm) and long-lived plasma cells (LLPCs). By modelling dynamic microarray gene expression data, Deng *et al.* proposed that Tfh-cells are generated initially in the spleen during *Bp* infection, and then migrate to the lung to participate in the GC reaction [123].

In humans, findings on the impact of different infant pertussis vaccines on the magnitude, maintenance and function of the Bm compartment are inconsistent and inconclusive, potentially reflecting the heterogeneity of the interrelated T-cell data [38,96,98,100,124,125,126,127]. More recently, significantly fewer B-memory cell populations were measured post-booster in older children who had completed aP versus wP infant immunisation [39]. Similarly, the effect on pertussis-specific antibody avidity indices varies depending on infant vaccine type, although results are conflicting [98,124,128]. These findings possibly indicate suboptimal germinal centre T-cell/B-cell interactions at priming and subsequent differences in vaccine-induced longevity and efficacy of protection. There is minimal data, however, on the role of vaccine-induced pertussis-specific LLPCs in long-term protection, largely due to the challenges in accessing these cells within the bone marrow where they primarily reside long-term; this is compounded by our limited understanding of LLPC phenotype and timing when they transit from secondary lymphoid organs to bone marrow [129].

Finally, although CD8^−/−^ mice remained protected following aerosol *Bp* challenge, a detailed characterisation of pertussis-specific CD8+ T-cell responses in humans remains unclear and would be informative [66]. This is given that *Bp* acts as both an intracellular and extracellular pathogen, as well as the importance of IFN-γ, a key cytokine also produced by cytotoxic cells. Rieber *et al.* quantified pertussis-specific CD8+ T-cell activation following adolescent aP booster, demonstrating an increase in peripheral CD8+CD69+ activated pertussis-specific memory T-cells four weeks after vaccination, regardless of infant priming [110]. Other studies in both convalescent and vaccinated participants have similarly identified pertussis-specific CD8+memory T-cells, which may contribute to protection against clinical pertussis [103,130].

### 3.6. The Human Challenge Model May Help to Address Gaps in Knowledge, Particularly on Mucosal Immunity

Recently, a first-in-human controlled infection model (CHIM) has been developed. Subjects were challenged by intranasal inoculation: the dose was gradually escalated from 10^3^ colony forming units (CFU) (none colonised) to 10^5^ CFU (80% colonised). At least a three-fold rise in serum anti-PT -IgG level was observed compared to baseline by day 28 in 9/19 colonised subjects but not in uncolonised participants [131,132]. This demonstrates that safe, deliberate induction of asymptomatic *Bp* colonisation is possible and induces a systemic immune response. Both blood and mucosal samples are being collected for analysis and results are urgently awaited. CHIM is a valuable tool to further explore *Bp* colonisation and associated immune responses in natural infection and vaccine development; nevertheless, due to ethical and safety concerns, challenge can only be achieved in adult cohorts. One possible practical solution in the paediatric age-group is to contextualise adult controlled-infection data within infant vaccine studies, linking mucosal responses induced by infection to those elicited by immunisation. Vaccines are a useful tool to probe the infant immune system, circumventing the need to challenge with the bacterium itself, as has been shown for viral pathogens such as influenza [133,134,135].

## 4. Measuring Vaccine-Induced Cellular Responses in Humans

### 4.1. How Do We Measure T-Cell Responses in the Blood?

Proliferation and clonal expansion of the antigen-specific T-cell population is crucial to orchestrating the development of protective immunity and immunological memory [136]. Defining vaccine-induced T-cell immunity, however, is complex and hampered by practical and biological challenges [137,138,139,140]. In humans, the primary source of T-cells is from the blood, which does not always accurately recapitulate immune events occurring in the tissue or mucosa. Furthermore, their very low frequency in peripheral blood means that easy enumeration and phenotypical characterisation of the total pool is difficult, requiring the processing of high cell numbers and/or highly specific analytical methods; frequencies of antigen-specific T-cells within the human memory repertoire are usually in the range between 10^−5^ and 5% [46]. This is further compounded by small sample volumes, particularly in infants.

Up until recently, limited parameters of the pertussis-specific T-cell response have been analysed, which may in part explain previous conflicting data. However, characterising the T-helper cell subset, memory phenotype, central/effector phenotype, homing/chemokine markers, cytokine release, T-cell repertoire and T-cell receptor sequence are all crucial to our understanding of the multidimensional pertussis-specific T-cell response.

#### 4.1.1. Traditional Methods

Two T-cell parameters have conventionally been measured: cell proliferation and/or cytokine production. The classical and most commonly used method to determine T-cell proliferative capacity is based on measuring titrated, radioactively-labelled thymidine ([^3^H]TdR) incorporation into the DNA of proliferating cells using a scintillation counter. The [^3^H]TdR assay is a simple and easy method to perform, with relatively high throughput; however, it requires radioactive facilities and specialised management of waste which may not be available [141]. An alternative is to determine incorporation of the pyrimidine analogue, 5-bromo-2′-deoxyuridine (BrdU) by peroxidase-labelled detection antibody using enzyme-linked immunosorbent assay (ELISA) [142].

The enzyme-linked immunospot (ELISpot) is also high throughput–friendly, conventional technique to quantify and measure antigen-specific T-cell responses to vaccine antigens at a single cell level that relies in their ability to produce cytokines in response to in-vitro antigen recall (after an overnight culture for effector memory cells or several days for central memory). It involves stimulating PBMCs with antigen(s) of interest seeded in tri- or duplicates on 96-well ELISpot plates coated with an anti-cytokine capture antibody. Cytokines produced by antigen-specific T-cells bind to the capture antibody and are detected using a biotinylated detection cytokine-specific antibody and enzyme-conjugated streptavidin and a chromogenic development substrate. This method is highly sensitive (cells producing fewer than 100 cytokine molecules can be detected) and the lower limit of detection can be under ten cytokine-producing cells per million PBMCs) [143,144]. Unlike an ELISpot assay, FluoroSpot can detect secretion of more than one cytokine simultaneously in the same sample using fluorescence instead of chromogenic detection [145].

The major disadvantages of these assays are limitations in the number of parameters to be investigated, lack of phenotypic information, and preferential detection of effector cells. When combined, this likely results in an underestimation of the total antigen-specific T-cell response. Moreover, the contribution of each cell population to the measured ELISpot/Fluorospot response is unclear.

#### 4.1.2. Flow-Cytometry Methods

Several flow-cytometric readouts for the direct detection of rare antigen-specific T-cells have been described in recent years (and used in pertussis vaccine studies), measuring (a) cell proliferation (b) intracellular cytokine release staining (ICS) (c) peptide-MHC-II tetramer staining (d) activation-induced markers (AIM). The advantages of these assays are that they allow co-staining with other markers, enabling identification of cellular sub-populations according to phenotypic and functional characteristics, amplifying the information to be acquired from a single readout [146].

(a) *Novel proliferation assays:* Classical proliferation assays have largely been replaced by flow-cytometric readouts. Examples include fluorescent dye dilution assays, using CFSE or derivative dyes, and assays that detect BrdU by fluorochrome-conjugated antibody staining [142,147]. Proliferation dyes diffuse easily into cells and bind covalently to the amino groups of intracellular proteins; since the dyes are divided equally between daughter cells the number of cell divisions of the proliferating cells can be visualised, thus allowing the theoretical enumeration of antigen-specific cells [136,141]. Expression of Ki67, a nuclear protein that coordinates regulation of cell division, is also increasingly being used to measure specific T-cell responses induced by pertussis vaccination, including longitudinal monitoring [148].
(b) *Intracellular staining (ICS)* is a versatile method used to analyse cytokine production at a single-cell level by flow cytometry, although it is less sensitive than ELISpot [149]. Blood or PBMCs are stimulated with the antigen(s) of interest and a transport inhibitor (e.g., BFA or monensin) is added for several hours to block secretion of the produced cytokines, thus allowing detection. Stimulated cells are then stained with fluorescently-labelled antibodies targeting surface markers then fixed, permeabilised and stained with anti-cytokine antibodies, before flow-cytometric analysis. This allows detailed phenotypic and functional analysis of pertussis-specific T-cell subsets and diverse differentiation pathways. Polyfunctionality, i.e., the ability of a cell to produce >1 cytokine, is also measured [150,151]. Technological advancements have enabled increasingly higher numbers of parameters to be assessed, although they must be pre-determined which can skew the response detected; given that standard panels in clinical trials often use IFN-γ, IL-2 and TNFα (and IL-4 can be difficult to detect), a more Th1-biased response may be established, which is particularly concerning when characterising vaccine-induced Th-immunity in pertussis vaccine research, in which establishing the ratio of different antigen-specific Th-profiles is important [138,152]. Furthermore, some memory cells may not secrete detectable levels of cytokines, especially the central compared to effector memory CD4+ population which develops at later time points after infection or vaccination [152]. Other drawbacks of this method include signal-to-noise ratio and nonspecific binding [139].
(c) *Peptide-MHC-II tetramer staining:* MHC tetramers are based on the structural features of the T-cell receptor and consist of 4 MHC molecules which are associated with a specific peptide/epitope [153]; the complex is bound to a fluorochrome, thereby enabling the direct visualisation, quantification and phenotypic characterisation of antigen-specific T-cells using flow-cytometry, following both pertussis infection and vaccination [139,153] This method, however, requires prior knowledge of the MHC alleles of the donor (particularly challenging in a LMIC) and relevant antigenic epitopes; moreover, it only gives limited and preselected insight into the heterogeneous T-cell populations specific for a certain antigen.
(d) *Activation-induced marker (AIM) assays:* More recently, techniques have been developed that do not require prior knowledge of MHC alleles nor focus on single cytokine responses but instead assess T-cell specificity irrespective of their functionality. Antigen-specificity is defined based on the upregulation of T-cell-receptor-stimulated surface markers, termed activation-induced markers (AIM), upon overnight antigen recall of either whole blood [154] or PBMCs [152,155], without the need for fixation and permeabilisation of cells for ICS.

Specific combinations of AIM markers used to detect antigen-specific CD4+ in pertussis vaccine research to date include CD40L (also termed CD154) alone or co-expressed with CD69 and, most commonly, co-expression of OX40 plus CD25; the latter has been used to identify the maximal antigen-specific response, with or without PDL1 to discriminate antigen-responsive T-reg cells which upregulate CD25 upon antigen stimulation, a potentially important compartment that may contribute to underlying immunological differences between aP and wP vaccines, as discussed previously [152,155,156,157,158]. In parallel, AIM combinations, such as CD107a and CD137 (4-1BB), or OX40 and CD25, have been used to identify antigen-specific CD8+ T-cells [156,159]. Comparisons of combinations have demonstrated that these assays have high concordance and identify distinct but overlapping populations of antigen-specific CD4+ T-cells; bystander activation was confirmed to cause minimal background [152].

Furthermore, as well as capturing the total pool of pertussis-specific T-cells, AIM assays are particularly beneficial in the identification of antigen-specific Tfh. This cell population is difficult to identify by cytokine production (ICS), given that their function is to primarily help adjacent germinal centre B-cells via cognate interaction, with limited cytokine secretion compared to other Th-cell types [155].

#### 4.1.3. Methods to Enrich Antigen-Specific T-Cells

Despite their significant advantages, these flow-cytometric technologies are limited by the number of cells per sample that can be acquired in reasonable time (10^5^–10^6^) as well as by the natural and/or technological background of the assay, which is typically between 0.01 and 0.1%. Sufficient target events are required to be able to identify small subsets with high statistical significance [160]. As such, methods have been developed to further increase or enrich the antigen-specific T-cell pool.

Firstly, *in-vitro* re-stimulation and development of libraries of polyclonal expanded memory CD4+ T-cells enables greater sensitivity to detect and enumerate rare antigen-specific T-cells; however, extended culture may alter the phenotype or function of responding T-cells, particularly activation, memory and homing markers [161]. The most recent successful strategy, therefore, has been to assess responses *ex-vivo* (following short overnight stimulation), by using pools of different epitopes or peptides, so that the overall frequency of responding cells is enhanced. This approach is particularly effective when combined with ELISpots or AIM assays described above and is critical to analysing small volume samples. A megapool approach is applied which consists of deconvoluting responses to large numbers of peptides of overlapping sequences, previously successful in pertussis vaccine studies as well as, more recently, to interrogate T-cell immunity to SARS-CoV-2 [46,47,109,162].

Secondly, the rapid antigen-reactive T-cell enrichment (ARTE) approach utilises magnetic-bead enrichment of TCR-activated T-cells that have upregulated the costimulatory molecule CD154 (CD40L) to assess human antigen-specific CD4+ T-cells *ex-vivo*. This relies on magnetic labelling of cells prior to separation in a magnetic field [157,158,160]. A CD40-blocking antibody needs to be added into the culture to prevent downregulation of CD154 expression. Furthermore, this type of enrichment can be combined with tetramer technology and used to identify rare peptide MHC-multimer–labelled cells from large sample sizes [153].

#### 4.1.4. Higher Throughput Technologies: Multidimensional Flow or Mass Cytometry

Multicolour fluorescence-based flow cytometry is more user and equipment friendly than mass cytometry, with easier access to antibodies, and it allows recovery of the cells by single-cell sorting. Enhanced flow cytometry with imaging capabilities enables the determination of cell morphology, as well as the spatial localisation of the protein molecules within a single cell. Advances in both microfluidics and digital PCR have improved the efficiency of single-cell sorting and allowed multiplexed gene detection at the single-cell level [149].

Nevertheless, flow cytometry is inherently limited by the number of parameters that can be simultaneously analysed. Recently, the advent of mass cytometry by time-of-flight (CyTOF) has facilitated deep interrogation of a large number of single-cell protein expression simultaneously [163,164]. It is based on the concept of using heavy-metal isotopes to label antibodies followed by mass spectrometry as the readout, in lieu of fluorochromes and light detection. As such, it avoids significant spillover between detector channels, which is a key challenge in fluorescence flow cytometry. High-dimensional phenotypic data can be visualised by combining CyTOF with algorithms such as visualisation of stochastic neighbor embed (viSNE) and spanning-tree progression analysis of density-normalized events (SPADE) [140,163]. As the throughput on CyTOF is lower than conventional flow cytometry, it can be coupled with magnetic bead enrichment for the sensitive analysis of rare antigen-specific T-cells, based on their expression of CD154+/−CD69, as described previously [164].

#### 4.1.5. Omics’ Technologies

Accumulating data from existing technologies demonstrates that there is considerable, previously underappreciated, heterogeneity in T-cell responses; additional pathogen-related functional patterns emerge not necessarily associated with the dominant Th1/2/17 classification. Epitope-specific T-cells can be further characterised in-depth by transcriptomic profiling that uses deep-sequencing technologies, including bulk RNA-seq or single-cell RNA-seq(scRNA-seq) [46,146].

Transcriptome signatures for aP versus wP vaccine responses are being defined in both animal models and human studies, characterising both innate and adaptive immune compartments [109,165]. Multiple key factors in Th-differentiation, including transcription factors, cytokines, and receptors, have been identified within the differentially expressed genes [165]. A recent murine study aimed to elucidate the kinetics of the protective immune response evolving after experimental *Bp* infection in mice, including activation of particular transcription factors and cell markers; using a systems approach they provided detailed insight into molecular and cellular sequence of events connecting different phases (innate, bridging and adaptive) of the immune responses, detecting a prolonged acute phase response, broad pathogen recognition, and early gene signatures of subsequent T-cell recruitment in the lungs. In addition, signatures preceding the local generation of Th1 and Th17 cells as well as IgA in the lung were identified [166].

Example human studies include the characterisation of transcriptomic profiles of pertussis-specific CD4+ T-cells from aP- versus wP-primed donors, in order to shed light on the nature of the key differences between the infant vaccines [109]. Differential gene expression was noted which corroborated flow-cytometry findings at the protein level (including the upregulation of the IL-9 gene in wP versus IL-5, IL-13, and TGF-β genes in aP donors, described previously). Moreover, the team applied Gene Set Enrichment Analysis (GSEA), a computational method that determines whether certain biological functions are significantly represented in the input group of genes, as well as Ingenuity Pathway Analysis (IPA) to obtain additional functional insights; for example, this helped to establish that mitosis and cell-cycle progression genes that promote cell division were increased in wP samples. Their results suggested that aP versus wP priming is associated with alterations in specific T-cell subsets (as suggested by the differential polarisation) and in cell proliferation (consistent with lack of *in-vivo* boost in aP-primed donors) [109]. Furthermore, White *et al.* also used an unbiased systems biology approach to elucidate Th2-associated responses to aP infant vaccines via gene co-expression network analysis, which identifies genes that function co-ordinately in complex pathways, comparing to other vaccines. They demonstrated that following TdaP, potentially antagonistic Th1-/IFN-associated and Th2-associated gene networks coexist in an apparent state of dynamic equilibrium [167]. Next-generation OMV vaccine responses are being interrogated using a similar systems approach; subcutaneous and pulmonary immunisation routes were recently compared in mice, combining analysis of innate responses and induction of mucosal and systemic T and B-cell responses (including CD103+ T-cells) [168].

In comparison with bulk RNA-seq, scRNA-seq is a more powerful tool to investigate vaccine-induced cellular heterogeneity and identify novel subpopulations, by applying a granular and unbiased approach [169,170]. Combined TCR-transcriptome analysis, for example, has been applied successfully to interrogate yellow fever virus vaccine-reactive T-cells [171]. Delving further into the transcriptome of single-cells using RNA-seq is likely to reveal the fine-specificity of cellular events such as alternative splicing (i.e., splice variants) and allele-specific expression, and will also define the roles of new genes [46,146]. Detailed analysis of clonally-related antigen-specific T-cells using scRNA-seq provides information on pathways of differentiation of memory T-cells; using pseudotime tools, trajectories of T-cell differentiation can also be inferred [172,173]. These technologies have advanced substantially in the field of cancer immunology: vaccine research is just catching up.

### 4.2. How Do We Integrate This with Knowledge of T-Cell Epitopes?

#### 4.2.1. Epitope Mapping

An important aspect when interrogating antigen-specific T-cell populations is to characterise the corresponding antigen targets and most immunodominant T-cell epitopes. To date, T-cell epitopes elicited by either natural *Bp* infection or wP immunisation have not been comprehensively defined [53]. Indeed, one of the key problems in elucidating the differences between aP compared to wP vaccines has been the lack of an appropriate antigen to tease out antigen-specific cells. The most common antigens used have been either the 5 key proteins in the aP vaccine or pertussis lysate, although the latter induces strong non-specific background responses due to the presence of *Bp*-specific as well as common bacterial antigens and innate receptor triggering ligands [150]. More recently, ‘megapools’ of overlapping peptides derived from the aP vaccine have been developed, as described in Section 4.1.3 [37,109]. However, a reliable pool of immunodominant epitopes derived from the wP vaccine is yet to be established, although is currently in the pipeline, based on bioinformatic epitope predictions and a full-scale genome-wide mapping of memory T-cell reactivity (using *ex-vivo* AIM readouts) to *Bp* antigens in humans [47]. Indeed, a similar genome-wide mapping of T-cell responses to *Mycobacterial tuberculosis* antigens was recently accomplished successfully [46]. An alternative in-silico approach can be applied to identify and modify T-cell epitopes in pertussis antigens that are cross-reactive with human sequences (and potentially tolerance-inducing); proteins with reduced tolerogenicity have improved vaccine potency in preclinical models [174].

Defining novel targets associated with T-cell reactivity and understanding patterns of epitope sequence variation throughout the population is also of considerable interest given the hypothesis that the switch from wP to aP generated a response that is less diverse, thereby creating an opportunity for *Bp* to escape vaccine responses. For example, Bart *et al*. identified a total of 471 coding SNPs from pre-vaccination strains. Precise mapping of epitopes that are not also targets of immune responses and a comparison of mutation rates of epitopes versus non-epitopes for both vaccines will further elucidate if the observed genetic variability of circulating *Bp* strains is indeed a result of T-cell immune pressure [175].

Beyond characterising epitope patterns, studies to determine HLA restriction to certain epitopes, associated HLA polymorphisms and binding promiscuity (which may vary by participant group/setting), HLA binding affinity and analysis of epitope conservation would also be informative [46].

#### 4.2.2. Combine with T-Cell Receptor Sequencing

TCRs dictate the antigen-specificity of T-cells through their interactions with peptide presented within MHC molecules. Epitope-associated TCR repertoires can therefore be defined by TCR sequencing [146]; more recently, using scRNAseq, both alpha and beta chains can be sequenced simultaneously. This enables robust investigation of the common features of TCRs that are specific for a particular epitope and identification of determinants that may predict specificity [172]. Knowledge of key TCR sequences can subsequently be integrated with understanding the functional, phenotypic and transcriptomic T-cell profiles related to recognition of specific epitopes, particularly novel targets identified in wP vaccines, as compared to the ones currently included in the aP vaccine.

### 4.3. How Do We Measure T-Cell Responses in the Mucosa?

To date our knowledge of vaccine-induced mucosal immunity has relied on animal models, given the practical difficulties in studying the mucosal antigen-specific T-cell compartment in humans. Unfortunately, immunological findings in animals often fail to translate fully to humans.

Early studies optimising AIM T-cell readouts, specifically in the context of defining Tfh responses, compared results in both blood and lymphoid tissue from adult donors who were undergoing tonsillectomy [155]. A more common and less invasive method to sample mucosal cells is a nasopharyngeal wash, although the luminal cell population can vary significantly from intra-mucosal cell populations. Recently, nasal curettes or brushes have been used to collect epithelial cells from the inferior turbinate for culture, gene expression and phenotypic/functional analysis using flow cytometry [176]. However, these techniques are challenging and less well-tolerated in infants.

Instead, measuring cytokine and other soluble immune mediator levels in either nasopharyngeal wash or mucosal lining fluid (MLF) provides a useful surrogate of mucosal CMI [177]. MLF is produced by the nasal inferior turbinate (i.e., upper airway ciliated epithelium) and can be collected using a unique ‘Nasosorption’ device, consisting of an absorptive matrix which acts like blotting paper; it has been used previously with success in the context of allergens, pathogens, challenge models or vaccine studies [178,179,180,181]. Superior cytokine detection was confirmed with absorptive matrices compared to nasopharyngeal wash [176]. One limitation of using this cytokine-based approach, however, is that the secreting cells cannot be characterised, and the readout may be susceptible to background ‘noise’, including numerous confounding factors that shape the upper airway immune milieu.

Furthermore, recent interest in TRMs has led to an increased focus on homing markers to capture these mucosal cells following activation as they migrate to their tissue of interest [26,43,182]. To date, homing markers have been established for gut and lower respiratory tissue but less is known about the upper airway. CD69 and CD103 are thought to be key lung homing markers, however, CD69 is also upregulated following TCR activation (AIM) [26].

Finally, the CHIM will contribute immensely to our knowledge and understanding of *Bp*-specific mucosal immunity and how it correlates to systemic responses in humans, following different immune challenges.

## 5. Which Factors May Affect T-Cell-Mediated Immunity to Pertussis Infant Vaccinations?

Multiple factors may shape CMI to pertussis immunisation throughout infancy, childhood and adolescence and need to be taken into account when analysing CMI as well as designing and testing next-generation vaccines. A detailed characterisation of all potential influencing factors, however, is beyond the scope of this review but has been explored by other authors and in the context of different vaccines [183].

### 5.1. Vaccine-Related Factors

The type of pertussis vaccine delivery (mucosal versus parenteral), formulation (antigen type, strain, dose and adjuvant content), platform and schedule are all important in modulating the antigen-specific cellular compartment. Strategies to harness these factors to improve vaccine-induced responses will be discussed in the final section.

### 5.2. Host-Related Factors: Intrinsic (Innate)

#### 5.2.1. Age

Data on the effect of age on vaccine-induced T-cell responses are conflicting. On one hand, no significant difference was found between full-term and preterm infants in their ability to mount a robust and specific cellular immune response to the administration of the primary course of pertussis vaccines [184]. Other studies, however, have observed quantitatively lower and/or qualitatively suboptimal long-term cellular responses in vaccinated pre-term (especially under 31 weeks) compared to term infants [16]. Indeed, it is well established that immune ontogeny in early infancy has distinct developmental restrictions or functional adaptations, particularly when compared to older children [134,185]. Impaired CD4+ and CD8+ T-cell frequency and function is observed, such as after primary immunisation, with marked Th2 skewing. This can be explained by epigenetic processes, lower soluble factors, and suboptimal frequency and distinct functional capacity of antigen-presenting cells [133,186]. Diminished B-cell interaction with T-cells is also observed, which subsequently affects germinal centre responses and B-cell maturation [187]. Indeed, in comparison to term infants, preterm infants have significantly lower pertussis antibody levels following both completion of the primary immunisation course and, in some studies, even subsequent pertussis boosters [188,189,190]; this may be partly driven by T-cell dependent mechanisms.

#### 5.2.2. Sex

The vaccine response profile can vary according to sex, partially explained by differentially elevated levels of gonadotropins and sex hormones in male and female infants in the first few months of life, usually decreasing by 1-year of age [183,191]. Furthermore, a number of immune response genes and microRNAs are also encoded on the X-chromosome [192]. There is a paucity of data to date, however, on the impact of sex on pertussis vaccine immunogenicity.

#### 5.2.3. Genetics

Various ethnic groups living in the same location have different vaccine responses suggesting a genetic influence on vaccine responses [183,193]. Studies of twins estimated the degree of heritability following aP immunisation to be 53–65% for cellular responses, primarily IFN-γ and IL-13 induction [193,194,195]. Polymorphisms in critical genes significantly modulate CMI to infant vaccines, including major histocompatibility complex (MHC) genes as well as non-MHC factors, such as pattern recognition receptors, blood group antigens and genes involved in key immune signalling pathways [196,197,198,199,200]. For example, a Dutch infant cohort study demonstrated the association of a single-nucleotide polymorphism in TLR4 (role described in Section 2) with magnitude of PT-IgG following wP vaccination [201].

### 5.3. Host-Related Factors: Intrinsic (Acquired)

#### 5.3.1. Microbiome

Studies are increasingly showing a mutualistic relationship between vaccine responses and the intestinal microbiota, although data in humans is only recently emerging [202,203,204]. Vaccine-induced protection against *Bp* was reduced in azithromycin-treated mice, associated with impaired CD4+ T-cell memory, including lower numbers of lung TRMs and IL-17-production, as well as CD49d expression on splenic CD4+ T-cells after challenge [205]. Zhang *et al.* also showed that antibiotic-mediated dysbiosis in the murine intestinal microbiome resulted in immunomodulation, with increased susceptibility to *Bp* infection initially. This was associated with a significant deficiency in systemic Ig, IgG1 and IgG2a antibody responses as well as impact on the short-lived plasma-cell and Bm recall responses, CD4+ T-cell generation and PD-1 expression on CD4+ T-cells (likely perturbing plasma-cell differentiation) [206].

The contribution of the respiratory microbiome (nasopharynx and/or lung), however, is unclear [207,208,209]. There is minimal data in the context of pertussis immunization. One murine study on *Bp* infection demonstrated the important role of resident nasal microbiota; delivering broad-spectrum antibiotic treatment before *Bp* inoculation enabled it to efficiently colonise the nasal cavity, while subsequent reintroduction of single *Staphylococcus* or *Klebsiella* species in the nose of the same mice was sufficient to inhibit *Bp* colonization [210].

Furthermore, underlying immunological mechanisms remain elusive; changes in the level of microbially-derived metabolites may activate the innate immune compartment, further shaped potentially by breast milk components, thereby modulating development of T-and B-cells [203,211,212,213,214]. Pro *et al.* recently showed that the sequence homology between antigens and the human microbiome can either dampen (tolerogenic effect, most dominant) or increase (inflammatory effect) T-cell epitope immunogenicity; this occurs via molecular mimicry and is partially determined by bacterial genus. It is currently unknown whether *Bp* antigens and epitopes that share significant homology to human microbiota might be preferentially recognised or conversely tolerised and, therefore, how this might affect T-cell-mediated vaccine immunogenicity [215].

Conversely, changes in the local immune milieu induced by vaccines may shape the upper airway microbiome itself, thereby affecting the susceptibility against infective respiratory pathogens, potentially including colonisation and infection by *Bp* [216]. In the context of pneumococcal conjugate vaccines, different serotype coverage results in varying diversity and stability of the microbiota composition [216].

#### 5.3.2. Co-Morbidities and Malnutrition

The condition of the host, including primary and acquired immunodeficiency (e.g., HIV and immunosuppressive therapies), evolving infections (e.g., viruses, malaria, helminths) and chronic illness may impact vaccine-induced CMI [183,217]. Chronic CMV or EBV infections are prevalent in the first year of life, particularly in LMICs. The former was associated with very high frequencies of highly differentiated CD8+ and CD4+ T-cells in early life as well as a reduction in antigen-specific antibodies and limited memory CD4+ IFN-γ responses following measles vaccination [217,218]. Further work in the context of pertussis immunisation is warranted. Other studies, however, have found no association between low level of pertussis antigen-induced IFN-γ secretion and certain comorbidities, including very low infant birth weight, severe infections, corticosteroid treatment or the administration of gamma-globulins [184]. Furthermore, nutritional status (general malnutrition or vitamins A/D, zinc and iron levels) and the use of micronutrients at the time of immunisation may also play a role in the timing, quality and duration of infant vaccine responses [219,220,221,222]. Indeed, a 2014 Senegalese study demonstrated that birth season and undernutrition modulated children’s humoral response to pertussis toxin, which may be T-cell dependent, although CMI was not evaluated [223]. Data is heterogeneous, however, with generally poor quality of evidence [220].

### 5.4. Host-Related Factors: Extrinsic

#### 5.4.1. Interactions with Other Neonatal/Infant Vaccines

Recently, there has been growing interest in the ‘non-specific effects’ (NSE) of newborn, infant or even maternal vaccinations, whereby they may confer protection against pathogens unrelated to their original target and therefore have a broader impact on infant health [222,224,225]. Although not fully understood, two underlying immunological mechanisms have been proposed: ‘trained innate immunity’ and ‘heterologous immunity’. The former describes the ability of the innate system to generate immunological memory, mediated by epigenetic and metabolic reprogramming, and therefore be ‘trained’ to provide partial protection against subsequent infections [226,227]. The latter describes the effects on the adaptive immune system, primarily due to T-cell-mediated cross-reactivity between vaccine-related and -unrelated antigens [228,229]. Neonatal BCG, for example, has been hypothesised to provide an immune priming benefit across different settings, by modulating not only vaccine-specific responses but also heterologous T-cell immunity to off-target antigens (specifically Expanded programme on Immunisation [EPI] vaccines such as dTaP/dTwP) [230]. Moreover, several vaccines potentially alter antibody responses to unrelated antigens indicating an effect on B-cell function too.

Conversely, there is an ongoing debate whether neonatal or infant vaccines may in fact facilitate the development of immune cell hypo-responsiveness instead, particularly when challenged with either the same antigen (‘immune paralysis/tolerance’) and/or concomitant antigens (‘vaccine or bystander interference’) in subsequent infant immunisation schedules [134,228]. Potential interference will, therefore, need to be considered for any novel next-generation pertussis vaccines that may be introduced in the future, either alongside existing EPI vaccines or combined into multivalent formulations [231].

#### 5.4.2. Maternal Factors

Maternal pertussis immunisation programmes are being introduced globally, particularly in high-income settings [232]. Vaccine-induced maternal antibody is transferred across the placenta or in breast milk and may interfere with subsequent infant vaccine responses. Most findings to date have focussed on the impact on the infant humoral compartment, although underlying mechanisms are not fully understood and the clinical implications have yet to be fully elucidated, particularly given the lack of a CoP [233,234]. A recent 2017 meta-analysis combined the serological data from 32 randomised-control trials conducted in 17 countries on a total of 7630 infants, concluding that antigen-specific maternal antibody inhibits infant antibody responses to priming vaccines and that these effects could be long-term as they are not always abolished by the administration of a booster dose; this includes responses to PT, FHA and PRN [235]. A recent study, however, showed that maternal antibodies may similarly modulate vaccine-specific cellular compartment in infants, for example, by limiting the expansion of Tfh cells and thus dampening germinal centre output [236]. Findings are scarce and inconsistent, however, with other studies demonstrating that maternal vaccination does not affect natural infection- or vaccine-induced infant CMI [237,238].

Beyond this, evidence is now emerging that the fetal and subsequently infant immune system may be shaped by maternal vaccination through more than just the passive immunity provided via antibody transfer [232]. One possible—albeit poorly understood—mechanism is maternal ‘microchimerism’ (MMC), which occurs secondary to low frequency, bi-directional migration of cells via the transplacental (and possibly breast-milk) route; MMC may play a role in neonatal immunomodulation, particularly maturation of the T-cell response [239,240]. These cells express non-inherited maternal antigens and can persist in the offspring long-term, particularly in T-cell or TRM compartments [234]; it is estimated that up to 1 in 5000 PBMCs may be of maternal origin [241]. Similarly, limited animal experiments and few human-based observations suggest that maternal immune cells can also be detected in breast milk and may traffic to infant tissues through gut mucosae [242,243].

Secondly, the fetus may be sensitised *in utero*, both qualitatively and quantitively, to infection- and non-infection-derived antigens which the mother has encountered during pregnancy, either as free antigens or antigen-antibody complexes or, potentially, antigen-loaded vesicles [232,244,245,246,247]. This may alter infant immunity, susceptibility to later childhood infections, vaccine responses and development of immunopathological disorders. *‘In-utero* priming’ was first proposed in neonates born to infected mothers, who are exposed to pathogens but uninfected themselves [246,248]. For example, infants of helminth-infected mothers are exposed to parasite-derived products that seem to cross the placenta and potentially prime or tolerate the fetal immune response to parasite-specific as well as unrelated antigens [249,250]. This early exposure may have long-term effects on the child’s immune system, including their CMI to pertussis childhood vaccines [249]. To date, however, there has been no robust, consistent evidence that vaccine-induced (including pertussis) immune responses of infants are strongly suppressed or affected by the helminth infection status of the mother [249,251,252,253]. In fact, strongyloidiasis was associated with enhanced responses to PT [251]. Similarly, empiric treatment of mothers with antihelminths (e.g. albendazole or praziquantel) during pregnancy does not influence infant responses to pertussis or other EPI vaccines [249,251,252,253]. Other maternal infections, such as malaria or HIV, may also play a key role in eliciting immune dysfunction in young infants, particularly in LMICs [254]. Despite comparable pertussis-specific T-cell proliferation between the two infant groups, HIV exposure significantly diminished measurable CD4+ and CD8+cytokine polyfunctionality in response to *Bp* stimulation [255]. These differences may be due to *in-utero* perturbation of the TCR repertoire secondary to maternal antigen exposure, such as HIV and opportunistic pathogens, or anti-retroviral therapy, as well as chronic immune activation and inflammation [255,256,257].

Although controversial, *in-utero* priming may also occur following maternal immunisation during pregnancy, as suggested by the presence of vaccine antigen-specific B-cell and memory-T-cell responses in cord blood in some studies, assumed to be secondary to activation of the fetal adaptive immune cell compartment [258,259]. This has been demonstrated following maternal influenza and tetanus vaccination, although there is little data following pertussis immunisation. How this may relate to and modulate subsequent infant CMI to pertussis vaccines, both at the systemic and mucosal levels, is yet to be fully elucidated. Interestingly, maternal MF59-adjuvanted influenza immunisation was associated with an altered cytokine profile in the nasal mucosa of 4-week-old infants subsequently, when compared to those born to unvaccinated mothers [260].

Finally, the maternal peri/postnatal microbiome is also thought to contribute to shaping early infant immune development and function, including modulating metabolites that originate directly from the maternal diet and are transferred to the offspring, potentially through breast milk [261,262]. Moreover, a recent review concluded that maternal malnutrition and subsequent intrauterine macro- and micronutrient deficiency is likely to impair infant responses to vaccines, even in the presence of nutrient supplementation, although evidence is limited and/or average quality [263]. There are no current studies relating this complex interrelationship to subsequent infant vaccine responses (including pertussis).

## 6. The Future: How Can We Harness Our Knowledge of Cell-Mediated Responses to Improve the Next-Generation of Pertussis Vaccines?

### 6.1. Harnessing CMI to Improve Vaccine Testing

Determination of a reliable CoP is an important part of the vaccine development process and aids progress towards licensure [57]. To date, pertussis vaccine-induced protection has primarily been measured by ELISAs that quantify total PT-specific antibody titres [59]; assays that measure functional antibodies (e.g., opsonising, neutralising, serum-bactericidal) are also important, although poorly standardised [264,265,266]. Indeed, none of the current assays are gold standard yet and a clear CoP for pertussis immunity with a threshold value known to provide clinical protection remains to be established. Given that CMI plays a key role in the quality and duration of protective immunity to *Bp*, a CoP based on accurately enumerating and phenotyping T-cell immunogenicity would be informative for both preclinical studies and clinical trials, particularly if novel pertussis vaccines are to capitalise on the T-cell response [58,138]. Identifying appropriate T-cell parameters has so far been challenging without a clear understanding of the mechanisms that afford protection. One potential approach may be to determine separate co-CoPs against infection/colonisation versus symptomatic disease.

Important biological and logistical considerations when running these T-cell assays in vaccine trials include use of whole blood versus PBMCs; running fresh versus frozen samples; testing the type and concentration of stimulating antigen plus length of stimulation; setting up *ex-vivo* versus *in-vitro* readouts. Facilitating reproducibility, reducing interlaboratory variability (including operator, reagents and equipment differences across sites) and standardising and validating T-cell assays (including appropriate cell viability and positive controls) is crucial, notably in the context of multi-centre randomised control trials which may include poorly-resourced settings [267]. The ability to obtain T-cell readouts from cryopreserved cells is particularly important for logistical planning, because it enables the assay to be run across multiple centres and batching of samples from multiple time points, improving efficiency and reducing experimental variation (although some markers may be susceptible to freezing). Vaccine trial sample size calculations are also problematic, given the paucity of data on infant immunity and heterogeneity in immune responses, therefore requiring large numbers of recruits to reach statistical significance [7,140,268]. PERtussIS Correlates Of Protection Europe (PERISCOPE) Consortium was established in 2016 to address some of these obstacles, by enabling fruitful collaborations between key academic, public and private stakeholders; by generating robust technologies and infrastructure for future vaccine development; and by establishing novel, standardised, validated biomarkers of protective immunity [269].

Antigen-specific T-cell responses in multi-centre vaccine trials are routinely determined using cytokine-based approaches, such as ELISpot and ICS assays described previously, avoiding the need for labour-intensive HLA typing. A recent pertussis vaccine study also characterised the cytokine profile in culture supernatant (using a multiplex Luminex assay) collected following 24–48 h whole blood stimulation with antigen [150]; although the exact cell-type secreting the cytokines cannot be ascertained, findings correlated well with flow-cytometric readout of ICS run in parallel. This type of supernatant assay may therefore provide a practical, easily reproducible alternative that can be applied even in low-technology settings. However, cytokine-based methods likely underestimate the frequency of the antigen-specific response and their limitations have been described previously in Section 4.1.

By contrast, the *ex-vivo* AIM assay provides a comprehensive account of the total antigen-specific response and could therefore be a valuable tool to establish a clearer immunological CoP. Practically, AIM assays require only short stimulation, small blood volumes and can be used with cryopreserved PBMCs. Bowyer *et al.* have provided the first in-depth assessment of vaccine-specific CD4+ and CD8+ T-cells using AIM panel OX40, CD25, PDL1 within a clinical trial setting [152]; in comparison to their ICS readout, the limit of detection was significantly lower with 2–3 times more antigen-specific T-cells detected at the peak time point [152]. Further validation, however, is required for the use of the AIM assay as a primary immunogenicity measure within phase II/III clinical trials of future vaccine candidates. Recently developed molecular techniques are also being used to assess immunogenicity in pre-clinical studies, with the potential to be applied to very small blood volumes in clinical trials as possible CoP. However, their high costs and requirement for state-of-the-art equipment and expertise currently precludes their widespread use, particularly in LMICs.

By combining different technologies, we can dissect the frequency, phenotype, and function of pertussis-specific T-cells in greater detail than previously possible. Flow or mass cytometric readouts can be combined: for example, AIM/ARTE or MHC tetramers together with other phenotypic surface markers and ICS. This can further be coupled with single-cell sorting followed by functional assays and/or transcriptomic or TCR analysis. Longitudinal characterisation of pertussis vaccine-specific T-cell immunity needs to be done in parallel with profiling and kinetics of the B-cell, humoral and innate responses. The data that emerges can subsequently be interrogated using bioinformatic analytical tools, including machine-learning-based gating algorithms for flow cytometry readouts [170,270,271]. It is important that relationships are analysed in a biologically plausible way and that data is collected and combined from different immune interfaces i.e., both peripheral blood and mucosal samples. A recent systems vaccinology approach combining transcriptomic, proteomic, cytometric and serologic profiling revealed that, while broad immune signatures to TdaP booster are shared between aP- and wP-primed individuals, a subset of aP-primed individuals show divergent response patterns [272]. Furthermore, data on factors that may affect CMI (discussed in Section 5) should be collected and included in the final efficacy analysis of pertussis vaccine studies or trials, especially if multiple centres across settings with differing population cohorts/demographics are involved [267,268]. If found to be play a significant role, these factors can potentially be modified or interventions introduced to improve vaccine immunogenicity, efficacy and/or longevity. Analyses must also consider age-related changes in longitudinal cohorts and include identification of preterm babies [273].

### 6.2. Harnessing CMI to Improve Vaccine Design and Delivery

Multiple strategies have been proposed to harness emerging knowledge of pertussis-specific vaccine cellular responses in order to develop the next-generation of improved, longer-lasting infant vaccines. These have been reviewed in-depth elsewhere but are summarised in Table 1 [1,7,26,43].

Above all, given that pertussis is acquired through the respiratory tract, nasally administered vaccines that stimulate mucosal immunity may prove more efficacious than vaccines eliciting solely systemic responses [274,275]. A variety of intranasal pertussis vaccines have been investigated in recent years along with different adjuvants; the most promising options include OMV or the live-attenuated BPZE1 vaccines, shown to effectively induce antigen-specific mucosal CMI and confer protection against both nasal and lung colonisation (Table 1).

## 7. Conclusions

**Figure 2:**
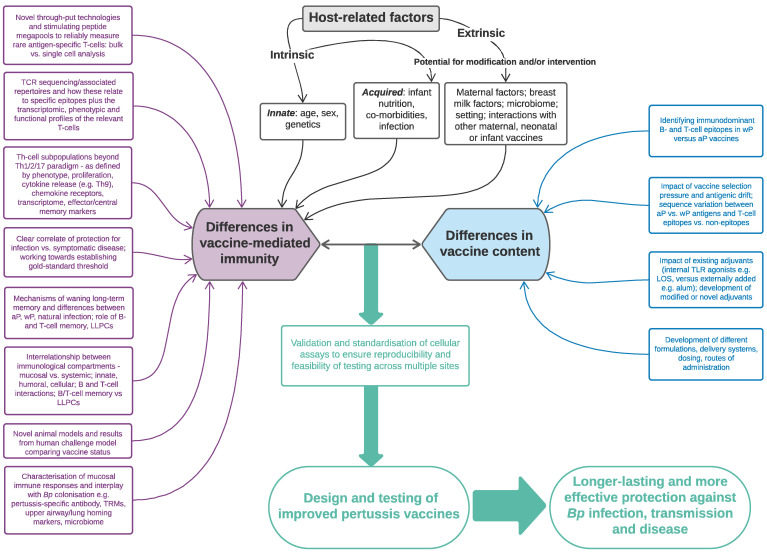
Schematic summary of future research priorities to characterise the differences between acellular and whole-cell pertussis vaccines, ultimately aiming to improve the design and testing of longer-lasting and more effective next-generation pertussis immunisations. Key gaps in knowledge are outlined which shape our understanding of differences in either vaccine-mediated immunity or vaccine content, both of which are interdependent and interacting. aP, acellular pertussis; *Bp*, *Bordetella pertussis*; LLPC, long-lived plasma cell; LOS, lipooligosaccharide; Th, T-helper; TLR, Toll-like receptor; TRM, tissue-resident memory; wP, whole-cell pertussis.

The switch from wP to aP vaccines in the primary infant immunisation schedule is increasingly considered the most plausible explanation for pertussis resurgence, although enhanced surveillance and more accurate diagnostic tools have also contributed to the recent increase in pertussis reporting. Neither vaccine induces the same robust, long-lasting protection as natural infection, with aP-mediated immunity waning far more rapidly. Moreover, a growing body of evidence from animal models suggests that aP vaccines fail to prevent upper airway colonisation and induce sterilising mucosal immunity, thereby facilitating *Bp* transmission in fully vaccinated populations by asymptomatic aP-immunised carriers. This phenomenon is also compounded by aP vaccine-driven emergence of *Bp* strains that have modified or absent epitopes, particularly pertactin. Understanding the underlying mechanisms of waning immunity and inadequate protection against infection/transmission, both at the systemic and local mucosal levels, will guide the design and testing of novel, effective immunisation strategies. The ideal pertussis vaccine would be administered as one dose intranasally in infancy, eliciting robust, broad and durable mucosal (and systemic) immunity, including pertussis-specific sIgA(/IgG) alongside Th1/Th17-polarised TRMs. Protection would thereby be conferred against lung disease as well as nasopharyngeal colonisation and subsequent transmission. Lessons are being drawn from previous animal models and human vaccine studies/clinical trials and, more recently, the controlled human challenge model. A set of assays that effectively and reliably capture antigen-specific T-cell responses should accompany these evaluations. Standard T-cell assays measure cell proliferation and/or cytokine secretion, while a new generation of sensitive and/or high-throughput methods have facilitated direct analysis and characterisation of rare antigen-specific cell sub-populations, particularly multidimensional flow cytometry, CyTOF and transcriptomics/genomics technologies. Single-cell analyses have focused on measuring the quality and breadth of responses, which must be combined with rigorous mapping of immunodominant T-cell epitopes, as yet unknown but in the pipeline for wP vaccines. Figure 2 summarises the key gaps in knowledge and avenues for future research to understand the differences between aP and wP vaccines.

## Figures and Tables

**Table 1:** Strategies to improve or guide novel infant pertussis vaccine design, including T-cell responses measured and protection conferred.

**Strategy**	**Examples**	**Vaccine-Induced T-Cell Responses**	**Protection Induced**
**Vaccine Delivery**			
***Intranasal*** ***(+/− adjuvant)***	BPZE1 (live-attenuated) vaccine and derivatives (e.g., BPZE1f3) ▪ Immunogenicity confirmed in mice, baboons, rhesus macaques and humans [276,277,278,279,280,281] ▪ Currently in clinical trials (NCT03942406 and NCT0354149).	▪ Dominant Th1 response: TNF-α or IFN-y. ▪ Minimal Th2 (IL-13) or Th17 (IL-17A) cells detected in humans vs. Th17 responses in animals restricted to lung, spleen, or nasal cavity ▪ Some BPZE1–specific CD4+ T-cells expressed >1 cytokine indicating polyfunctionality. ▪ No induction of BPZE1–specific CD8+ T-cells ▪ Increase in circulating T-follicular-helper (cTfh) cells with expansion of activated PD-1+ICOS+ cells exclusively amongst Th1-type CXCR3+ cTfh	▪ Protection against both lung and nasal colonisation in mice (protective mechanisms differ). ▪ IL-17-dependent sIgA-mediated mechanism of BPZE1-induced protection against *Bp* nasopharyngeal colonisation in mice. ▪ Await results in humans
	GamLPV (live-attenuated) vaccine ▪ In humans	▪ Await results.	▪ Currently in phase ½ clinical trials (NCT04036526).
	Outer-membrane vesicle (OMV) vaccines [282,283,284] ▪ In mice	▪ Induces strong mucosal immunity, including Th1/Th17-polarised TRM responses in lungs ▪ Enhanced pulmonary/nasal IgA levels (mainly directed against Vag8 and LOS).	▪ Protection against both lung and nasal colonisation, potentially preventing transmission, in mice.
	Aerosol delivery of wP vaccine, no adjuvant [285] (in humans)	▪ T-cell responses not assessed ▪ Systemic and mucosal antibody induction described only	▪ Not assessed
	aP only or combined with IRI-1501 ▪ Purified whole ß-glucan particle derived from *Saccharomyces cerevisiae* [286] (in mice)	▪ Compared to convalescent mice, elicited a superior humoral immune response but poorer Th1/Th17 immune response	▪ Protection against high *Bp* burden in upper and lower respiratory tracts of mice ▪ Reduced markers of inflammation
	aP mixed with curdlan ▪ 1,3 ß-glucan derived from *Alcaligenes faecalis* [287] (in mice) ▪ Promotes vaccine localisation	▪ Curdlan binds to dendritic cells leading to a nuclear factor κB-mediated Th17 response ▪ No increase in TRM cells observed in the lung ▪ Increased IL-17; intranasal mucosal IgA and serum IgG response	▪ Adding curdlan does not improve respiratory *Bp* clearance compared to aP vaccination only
	aP mixed with genetically detoxified *Escherichia coli* heat-labile toxin (LT) ▪ Two mutants: LTK63, lacks ADP-ribosylating activity and LTR72, partial activity (in mice) [288]	▪ Enhance antigen-specific serum IgG, secretory IgA, and local and systemic T-cell responses, ▪ LTK63 promotes a mixed Th1/2 profile ▪ LTR72, especially at low dose, selectively enhances Th2 cells and high IgA and IgG titers.	▪ High level of protection against *Bp* burden in the lungs in mice.
	aP mixed with LP-GMP, comprising c-di-GMP, an intracellular receptor stimulator of interferon genes (STING) agonist, and LP1569, a TLR2 agonist from *Bp* (in mice) [42]	▪ Synergistically induces production of IFN-β, IL-12 and IL-23, and maturation of dendritic cells. ▪ Induces potent *Bp*-specific Th17 responses and IL-17-secreting respiratory TRMs	▪ Sustained sterilising immunity against *Bp* nasal colonisation as well as lung infection for at least 10 months.
	Bacterium-like particles (act as adjuvant) carrying pertussis antigens (in mice) [289]	▪ T-cell responses not assessed ▪ Antigen-specific IgG and IgA induced in mice	▪ Protection against high *Bp* burden and histopathological injury in lungs of mice
***Cutaneous***	aP with genetically detoxified PT (PTgen) administered using Viaskin^®^ epicutaneous patches on days 0 and 14, followed by dTaP on day 42 (in humans) [290]	▪ Targets antigens to Langerhans cells ▪ Increased anti-PT IgG and neutralising antibodies ▪ T-cell responses not directly assessed	▪ Not assessed
	Outer membrane vesicle (OMV) vaccine [282,283,291] ▪ Non-replicating (in mice)	▪ Induces a broad antibody and mixed systemic Th1/Th2/Th17 response against multiple antigens ▪ Poor induction of mucosal immunity and respiratory TRMs.	▪ Protection against *Bp* burden in lungs ▪ No protection against nasopharyngeal colonisation.
**Vaccine regimen**			
***Additional*** ***neonatal dose followed by routine infant schedule***	Administered as dTaP or aP (in humans) [292,293]	▪ *Monovalent aP*: T-cell responses not assessed. But ‘bystander’ interference with concomitant antigen responses (e.g., Hib, diphtheria, tetanus) possibly due to strong pertussis T-cell responses interfering with subsequent induction of Th-cells. ▪ *dTaP*-T-cell responses not assessed specifically.	▪ Unclear benefit
***Priming dose followed by homo- or heterologous booster***	wP primary immunisation followed by booster dose of aP or new vaccine (in humans) [294]	▪ T-cell responses not assessed specifically ▪ Mathematical modelling	▪ Pertussis incidence reduced by up to 95% ▪ 96% fewer infections in neonates
	*Prime and pull:* Systemic priming followed by nasal boosting [295]	▪ Potential induction of systemic then local (via TRMs) memory immune response against *Bp*	▪ Not investigated in the context of pertussis to date.
**Vaccine type/platform**			
***Live-attenuated***	BPZE-1 (in mice, non-human primates, humans) [276,277,278,279,280,281]	▪ As above	▪ As above
***Outer-membrane vesicle (OMV)***	Intranasal, pulmonary, subcutaneous or intraperitoneal types (in mice) ▪ Contain multiple *Bp* antigens and can be prepared as stable freeze-dried formulations) [168,282,283,284,287,291,296,297,298,299]	▪ Long-lasting Th1/Th17 responses elicited ▪ Inbuilt adjuvant: helps to activate inflammatory pathways in murine or human macrophages ▪ Intranasal and pulmonary delivery in mice induces mucosal immunity, including respiratory INF-γ- and IL-17-secreting TRM cells and IgA (not induced following subcutaneous route)	▪ All induce long-lasting immunity and protection against *Bp* burden in lungs ▪ Only intranasal delivery protects against upper airway colonisation ▪ Higher protective capacity against PRN(-) bacteria compared to aP.
***Particulate antigen***	Micro/nanoparticles (e.g., presenting PT) made from the biodegradable polymer poly(lactide-co-glycolide acid) (PLGA) [300,301]	▪ Induces Th1/Th17 response	▪ Confers protection against *Bp* burden in lungs and trachea of mice
	Liposomes e.g., H56/CAF01 subunit vaccine [302]	▪ Studies in *Mycobacterium Tuberculosis (Mtb):* elicited cells expressing high IL-2 and IL-17A Memory CD4+ T-cells efficiently homed into the lung parenchyma chronically infected with *Mtb.*	▪ Confers sustained protection against *Mtb* in mice ▪ Not yet investigated in the context of *Bp* in mice or humans to date
***Novel human vaccine types***	Recombinant vector vaccines and nucleic acids (mRNA, DNA) vaccines (e.g., developed against SARS-CoV-2 [303,304])	▪ *mRNA SARS-CoV-2 vaccine* - Th1-responses with RBD-specific CD8^+^ and CD4^+^ T-cell expansion. ▪ Positive correlation between RBD-specific CD4+ T-cells and IgG/neutralizing antibodies to RBD. ▪ *Adenovirus vector SARS-CoV-2 vaccine*- Th1-biased response and production of IgG1 and IgG3.	▪ Not investigated in the context of *Bp* to date.
**Vaccine formulation**			
***Improve existing*** ***antigens***	Example includes recombinant PT mutants e.g., NCT01529645; NCT02382913 (in humans) [305]	▪ CMI only assessed in small subset ▪ Response induced by principally Th-1-like ▪ Weak T cell-specific responses against PT	▪ Await further investigation
***Add novel antigens to*** ***aP vaccines***	Increase number of components in aP vaccines containing novel immunodominant epitopes (in humans) [102,306]	▪ T-cell immunity not directly assessed	▪ 2–3 component vaccines less effective against disease (especially mild) than 5 component or wP
	Recombinant Adenylate cyclase toxin—*Bp* virulence factor (in mice) [307,308](e.g., detoxified)	▪ Induces toxin-neutralising antibodies. ▪ Augmented mixed Th1/Th2 response	▪ Significantly reduced *Bp* load in the lungs compared to aP alone
	Add autotransporters e.g., Bvg-activated autotransporters; BrkA; Vag8 and SphB1 (via intraperitoneal or subcutaneous route in mice) [309,310,311]	▪ *aP + BrkA:* T-cell immunity not directly assessed. Antibody profile (IgG2a versus IgG1) indirectly suggests BrkA may not affect Th1/Th2 balance ▪ *aP + SphB1 and Vag8:* T-cells not assessed. ▪ Strong opsonising antibody responses	▪ Significantly reduced *Bp* load in the lower respiratory tract ▪ No reduction in bacterial load in the nasopharynx
***Increase antigen dose***	Increase amount of fimbriae (Fim2 and Fim3) in licensed 5-component aP (in mice, [312]; in humans, [102,306]) ▪ Fim may play a role in infection	▪ T-cell immunity not directly assessed in humans ▪ Preferential induction of Th1 response (IFN-γ) in response to the PT and FIM antigens	▪ Enhance vaccine efficacy against *Bp* lung infection without increasing reactogenicity
	TLR2 agonists e.g., BP1569 or a synthetic lipopeptide derivative LP1569 (in mice, [313]);cyclic dimeric guanosine monophosphate (CDGM) with *Bp* TLR2 agonist (in mice [42])	▪ *BP1569* activates murine dendritic cells and macrophages and human mononuclear cells ▪ *LP1569* enhances Th1, Th17, IgG2a responses ▪ *CDGM*: intracellular receptor stimulator of IFN genes; promoted Th1/17 responses; induces IFN-β, IL-12, IL-23 and maturation of dendritic cells	▪ *LP1569/BP2569:* superior protection against *Bp* burden in the lung than aP alone; protect against tracheal colonisation *CDGM:* protection against nasal colonisation
	LOS analogues/TLR4 agonists e.g., monophosphoryl lipidA (MPL) or LpxL2 from *Neisseria meningitidis* added to aP vaccine (in mice, [314])	▪ Little endotoxin activity. ▪ Reduced eosinophilia in lungs, reduced ex-vivo production of IL-4 by bronchial lymph node cells and IL-5 by spleen cells, suggesting reduced type I hypersensitivity (driven by Th2 response).	▪ Enhance protection against *Bp* colonisation in the lungs (vs. alum)
***Add novel adjuvants (to replace alum)***	TLR7 agonist e.g., SMIP7 (in mice, [315]); synthetic TLR7 agonist combined with PLG nanoparticles within aP (in mice, [301])	▪ Promote Th1/17 polarisation and Ig2a antibody responses	▪ Enhance protection against *Bp* burden in the lungs
	TLR9 agonist e.g., CpG oligodeoxynucleotides (in mice, [67])	▪ Promotes Th1/17 polarisation, Ig2a and IgG2c antibody responses	▪ Enhance protection against *Bp* burden in the lungs (vs. alum)
	*Bordetella bronchiseptica* colonization factor A (BcfA) added to alum in aP (in mice) [316]	▪ Modifies/attenuates alum-induced Th2 responses ▪ Promotes Th1/Th17 polarisation. ▪ Increased IL-17, reduced IL-5, increased ratio of IgG2:IgG1 antibodies	▪ More rapid bacterial clearance from the lungs than aP alone

Note: The T-cell responses measured may not necessarily correlate with the protection conferred; further data await. aP, acellular pertussis; *Bp*, *Bordetella pertussis*; CMI, cell-mediated immunity; GMP, granulocyte-monocyte progenitor. Hib, *Haemophilus Influenzae* B; LOS, lipooligosaccharide; PLG(A), polylactide-co-glycolide (acid); PT, pertussis toxin; RBD, receptor-binding domain; SARS-CoV-2, severe acute respiratory syndrome coronavirus 2; sIgA, secretory IgA; SMIP, Small modular immuno-pharmaceuticals; Th, T-helper; TLR, Toll-like receptor; TRM, tissue-resident memory; wP, whole-cell pertussis.
